# Barefoot Plantar Pressure Indicates Progressive Neurological Damage in Patients with Human T-Cell Lymphotropic Virus Type 1 Infection

**DOI:** 10.1371/journal.pone.0151855

**Published:** 2016-03-21

**Authors:** Beatriz Helena B. Vasconcelos, Givago S. Souza, Tatiana G. C. P. Barroso, Luiz Carlos L. Silveira, Rita Catarina M. Sousa, Bianca Callegari, Marília B. Xavier

**Affiliations:** 1 Universidade do Estado do Pará, Belém, Pará, Brazil; 2 Núcleo de Medicina Tropical, Universidade Federal do Pará, Belém, Pará, Brazil; 3 Instituto de Ciências Biológicas, Universidade Federal do Pará, Belém, Pará, Brazil; 4 Universidade do Ceuma, São Luís, Maranhão, Brazil; 5 Instituto de Ciências da Saúde, Universidade Federal do Pará, Belém, Pará, Brazil; Centers for Disease Control and Prevention, UNITED STATES

## Abstract

**Background:**

The human T-Cell Lymphotropic Virus Type 1 (HTLV-1) is a retrovirus associated with neurological alterations; individuals with HTLV-1 infection may develop HTLV-1 associated myelopathy / tropical spastic paraparesis (HAM/TSP). Frequent neurological complaints include foot numbness and leg weakness. In this study, we compared the distribution of the body weight on different areas of the foot in HTLV-1 patients with HAM/TSP, asymptomatic HTLV-1 patients, and healthy individuals.

**Methodology:**

We studied 36 HTLV-1 infected patients, who were divided in two groups of 18 patients each based on whether or not they had been diagnosed with HAM/TSP, and 17 control subjects. The evaluation included an interview on the patient’s clinical history and examinations of the patient’s reflexes, foot skin tactile sensitivity, and risk of falling. The pressure distribution on different areas of the foot was measured with baropodometry, using a pressure platform, while the patients had their eyes open or closed.

**Main Findings:**

The prevalence of neurological disturbances—altered reflexes and skin tactile sensitivity and increased risk of falling—was higher in HTLV-1 HAM/TSP patients than in HTLV-1 asymptomatic patients. The medium and maximum pressure values were higher in the forefoot than in the midfoot and hindfoot in both HTLV-1 groups. In addition, the pressure on the hindfoot was lower in HAM/TSP patients compared to control subjects.

**Conclusions:**

The neurological disturbances associated with HTLV-1 infection gradually worsened from HTLV-1 asymptomatic patients to HAM/TSP patients. Baropodometry is a valuable tool to establish the extent of neurological damage in patients suffering from HTLV-1 infection.

## Introduction

The human T-cell lymphotropic virus type 1 (HTLV-1) is a retrovirus of the *Retroviridae* family that infects CD4 T lymphocytes and stimulates their proliferation [1]. The most severe consequences of HTLV-1 infection are adult T-cell leukemia / lymphoma (ATL) and HTLV-1 associated myelopathy / tropical spastic paraparesis (HAM/TSP) [1–4]. HAM/TSP is a progressive demyelinating disease affecting upper motor neurons; it is characterized by sensory and motor deficits more pronounced in the lower extremities, incontinence, and impotence [5–13]. Depending on the geographic location, 0.3 to 4% of HTLV-1 infected individuals develop HAM/TSP [14].

HAM/TSP progression includes the degeneration of the spinal cord columns, often the lateral columns and occasionally the anterior and posterior columns [11]. The thoracic segments of the spinal cord are typically the most severely affected [11]. Spinal cord histopathology revealed that inflammation is a prominent feature in HAM/TSP: perivascular and parenchymal lymphocytic infiltrates were found in the white and grey matter, and activated microglia and macrophages were observed in the white matter, along with degeneration and gliosis [11]. The spinal cord lymphocytic infiltrates contained a mixture of CD4+ and CD8+ T-cells at early stages of the disease, whereas CD8+ T-cells were predominant at later stages [11].

In patients affected by HAM/TSP, the pelvic girdle and the lower limbs muscles are damaged, leading to spastic gait and decreased gait velocity and dynamic balance [10,15–17]. Any physical or physiological impairment in the muscles controlling the distribution of the body weight load on an individual’s feet will severely affect the balance [18]. Therefore, the assessment of the standing balance is essential to the treatment of altered gait and dynamic balance in neurological patients [19], as the evaluation of foot pressure points can indirectly indicate changes in the motor-sensory interactions controlling the body weight load distribution.

The feet support the body weight, especially in the orthostatic posture. Bipedal support is given by the tuberosities of the calcaneus and the heads of the first and fifth metatarsi [20, 21]. The body weight of an individual in the standing position is supported by the foot plantar surface, and the amounts of pressure applied on different areas of the feet represent indirect indicators of the mechanisms used to maintain body posture and symmetrical distribution of the body weight load [22]. In the standing position, the body weight is distributed between the hindfoot and the forefoot, which support about the 57% and 43% of the body weight, respectively [23]. The baropodometric analysis uses resistive or capacitive sensors to measure electrical current flow or capacitance on a footboard while the individual is standing on it [24]. It maps the pressure load on the foot plantar surface and thus allows functional evaluations.

The study of plantar pressure and balance can be used to develop methods for the prevention, assessment, and treatment of pathological changes that hinder normal gait and affect the quality of life [25]. Patients affected by HAM/TSP with severe motor and sensory impairments in the lower limbs are likely to have an unbalanced distribution of the body weight load on their feet. However, detailed quantitative information about this condition is still lacking. Therefore, in this work we investigated and compared plantar pressure distribution on different foot areas in patients with HTLV-1 infection suffering from HAM/TSP, asymptomatic patients with HTLV-1 infection, and control subjects.

## Methods

### Subjects

This cross-sectional study was done at the Institute of Health Sciences, Federal University of Pará, Brazil. Written informed consent was obtained by all the study participants. All procedures were in accordance with the Declaration of Helsinki and were approved by the Ethics Committee for Research with Human Subjects, Institute of Health Sciences, Federal University of Pará (report #633.187). The subjects were diagnosed at and recruited from the Tropical Diseases Nucleus, the center for reference and assistance with HTLV-1 disease in the State of Pará. HTLV-1 infection was diagnosed by an infectologist physician according to the criteria established by the World Health Organization (WHO) [26], based on the patient’s clinical history and neurological evaluation and the results of laboratory tests: ELISA (Cambridge Biotech, Worcester, MA, USA), Western blot analysis (HTLV blot 2.4, Genelab, Singapore), polymerase chain reaction (PCR), or a combination of these. The subjects were first diagnosed at the Tropical Medicine Nucleus according to the above criteria, or at blood banks during the screening for blood donation.

Patients unable to remain in standing position without assistance or suffering from conditions that may also impair the normal plantar pressure such as other neurological disorders, diabetes, rheumatic diseases, and peripheral vestibular syndrome were excluded from the study. Subjects with physiological conditions (like pregnancy) that could alter the normal plantar pressure were also excluded.

Thirty-six HTLV-1 seropositive individuals were divided in two groups of 18 patients each: the subjects in the HTLV-1 group did not show any clinical conditions compatible with HAM/TSP, whereas the subjects in the HAM/TSP group met the WHO criteria for HAM/TSP diagnosis. In addition, 17 age-, sex-, and body mass index (BMI)-matched volunteers were enrolled in the control group. Table 1 summarizes the characteristics of the study participants. There were no statistically significant differences in the weight, height, and BMI in the three groups. The patients in the HTLV-1 group had been HTLV-1 seropositive significantly longer than the patients in the HAM/TSP group (p < 0.05).

**Table 1:** Characteristics of the subjects in the three experimental groups.

	Control	HTLV-1	HAM/TSP
Sex	13 F, 4 M	14 F, 4 M	10 F, 8 M
Age (years)	44.8 ± 10.5	46 ± 11.7	51 ± 9.7
Weight (kg)	68.5 ± 18.2	65 ± 11.2	62.7 ± 8.1
Height (m	1.57 ± 0.1	1.55 ± 0.08	1.57 ± 0.07
BMI	27.2 ± 5.4	26.8 ± 3.8	25.3 ± 3.3
Time since diagnoses (months)	-	74.2 ± 74.8	67.4 ± 48.3*

*p < 0.05. F, female. M, male. BMI, body mass index. Data are presented as mean ± standard deviation (SD).

Additional clinical parameters such as reflexes, skin tactile sensitivity, and balance were also assessed. Patellar and Achilles reflexes were tested using a reflex hammer. Four HTLV-1 and nine HAM/TSP patients had altered Patellar and Achilles reflexes, and two HAM/TSP patients had altered patellar reflex.

The tactile sensitivity of the foot plantar skin was investigated using Semmes Weinstein monofilaments. We used 5 monofilaments with force levels of 0.2, 2, 4, 10, and 300 g. The patients were lightly touched with the monofilaments in 8 different foot areas (6 areas in the forefoot, 1 in the midfoot, and 1 in the hindfoot) and asked whether they felt the monofilament touching their skin. Each monofilament was used three times, starting with the lightest. The sensitivity threshold was defined as the lightest monofilament identified by the subject; whenever the threshold was higher than 0.2 g, the skin tactile sensitivity in that area was considered altered. Altered sensitivity in the forefoot, midfoot, or hindfoot was observed in respectively 13, 14, and 15 HTLV-1 patients, and in 16 HAM/TSP patients for each area.

Berg balance scale was used to evaluate the balance. Postural transfers, stationary balance during sitting and standing positions, functional reach, rotational components, and base of support were assessed through 14 tasks. The total possible score of the scale is 56, and the risk of falling was classified as low (scores > 41), medium (scores from 21 to 40), or high (scores < 20). All HTLV-1 subjects had a low risk of falling, whereas HAM/TSP patients had low, medium, or high risk of falling (9, 8, and 1 patient, respectively).

### Plantar Pressure Measurements

Barefoot plantar pressure measurements were performed using the capacitive pressure platform EPS/R1 (Loran Engineering, Castel Maggiore, Bologna, Italy), with 2 224 sensors distributed in 48 cm² and connected to a computer with Biomech software. Environmental illumination and sound conditions were kept constant during the evaluation of all subjects. The static analyses were performed with the individuals standing barefoot, with their feet apart at a distance proportional to the shoulders distance and their arms lying along the body. The subjects directed their gaze to a white circle target painted on the wall 1 m away. The plantar pressure was recorded in 6 sessions of 1 min each, 3 in the “open eyes” condition and 3 in the “closed eyes” condition. A 30 to 60 s rest interval separated two consecutive recording sessions. For further data analyses, the mean values calculated from the three sessions recorded in each condition were used.

The medium and maximum plantar pressure values recorded in ten different regions of each foot were reported; the ten areas were the hallux—toe 1 (T1), the four other toes grouped together (T2-5), all five metatarsi individually (M1, M2, M3, M4, and M5), the midfoot (MF), and the medial and lateral heel (MH and LH, respectively). In each trial, the pressure on each area was calculated as percentage of the total pressure on the foot.

Kruskal-Wallis test with Dunnet post-hoc test was used to compare the pressure measured in each foot region among the three groups (HTLV-1, HAM/TSP, and control). We also calculated the groups odds ratio to increase or decrease the medium and maximum pressure on the forefoot (T1, T2-5, M1, M2, M3, M4, and M5) and hindfoot (MH and LH). Each group was compared with the other two; the odds ratios were calculated for different forefoot and hindfoot percentage pressure values. We reported the highest percentage values associated with statistically significant odds ratios. We considered as statistically significant a p-value < 0.001 after adjustment for multiple comparisons. All statistical analyses were performed using BioStat 3.0 software [27].

## Results

**Fig 1:**
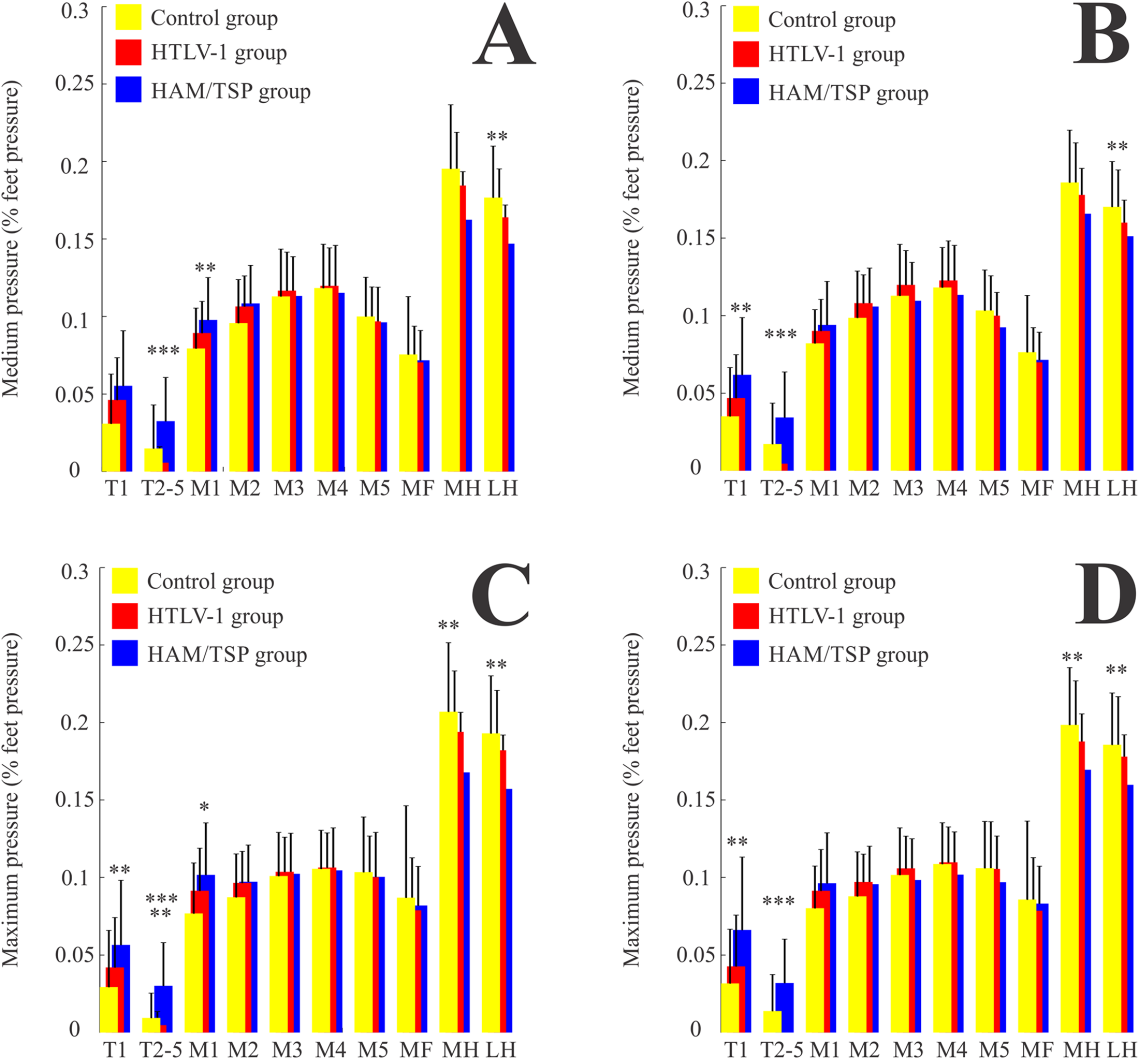
Plantar pressure distribution. Means and standard deviations of the medium (A and B) and maximum (C and D) pressure values obtained in the open eyes (A and C) and closed eyes (B and D) conditions in the three groups (control, yellow; HTLV-1, red; and HAM/TSP, blue). *p < 0.001 compared to both control and HTLV-1, **p < 0.001 compared to control, and ***p < 0.001 compared to HTLV-1.

Fig 1 shows the mean medium and maximum pressure values in different foot regions in the open eyes (panels A and C) and closed eyes (panels B and D) conditions for the three groups studied (control individuals, HTLV-1 seropositive asymptomatic patients, and HAM/TSP patients). In the open eyes condition, the medium pressure values in the HAM/TSP group were higher than the control group’s values at M1 (forefoot, p < 0.001), higher than HTLV-1 group’s values at T2-5 (forefoot, p < 0.001), and lower than the control group’s values at LH (hindfoot, p < 0.001). There were no statistically significant differences among the three groups in the medium pressure values at T1, M2, M3, M4, M5, MF, and MH. The maximum pressure values of the HAM/TSP group were higher than the control group’s at T1 (p < 0.001), higher than both control and HTLV-1 groups’ at T2-5 (p < 0.001), lower than both control and HTLV-1 groups’ at M1 (p < 0.001), and lower than the control group’s at MH and LH (p < 0.001). There were no statistically significant differences among the three groups in the maximum pressure values at M2, M3, M4, M5, and MF.

In the closed eyes condition, in the HAM/TSP group both the medium and the maximum pressure values were higher than the corresponding values in the control group at T1 (p < 0.001), higher than the corresponding values in the HTLV-1 group at T2-5 (p < 0.001), lower than the corresponding values in the control group at LH (p < 0.001), and finally the maximum pressure values were lower than the control group’s at MH (p < 0.001). We found no statistically significant differences among the three groups in the medium or maximum pressure values at M1, M2, M3, M4, M5, and MF.

The means and standard deviations of the medium and maximum pressure values in each group are shown in Table 2. We observed that the HAM/TSP group generally had higher values in the forefoot and lower values in the hindfoot compared to the other groups, whereas the HTLV-1 group generally had pressure values intermediate between the values of the control and HAM/TSP groups.

**Table 2:** Mean medium and maximum pressure values in different foot regions.

	Medium pressure (% total)			Maximum pressure (% total)		
	Control	HTLV-1	HAM/TSP	Control	HTLV-1	HAM/TSP
**Open eyes**						
**Forefoot**	55 ± 8	58 ± 7	61 ± 6*	51 ± 10	54 ± 8	59 ± 7*
**Hindfoot**	37 ± 7	34 ± 6	30 ± 6*	40 ± 8	37 ± 8	32 ± 7*
**Closed eyes**						
**Forefoot**	56 ± 7	59 ± 7	61 ± 5	53 ± 8	55 ± 8	58 ± 7*
**Hindfoot**	35 ± 6	33 ± 7	31 ± 8*	38 ± 7	36 ± 8	32 ± 7*

*Statistical difference between HAM/TSP group when compared to the control group.

Thus, we observed that the HAM/TSP group had a higher body weight load on the forefoot and less support on the hindfoot compared to the control group. Under physiological conditions, the percentage of the body weight load supported by the forefoot and the hindfoot is about 40% and 60% of the total pressure, respectively [23]. We observed that in the open eyes condition, compared to the control group, the HAM/TSP group was 5.26 times more likely to have a medium pressure value of 65% on the forefoot and 10 times more likely to have a maximum pressure value of 65% on the forefoot. In addition, compared to the control group, the HAM/TSP group was 16.6 times less likely to have a medium pressure value of 45% on the hindfoot, and 9 times less likely to have a maximum pressure value of 45% on the hindfoot. 65% and 45% were, respectively, the highest percentages of plantar pressure on forefoot and hindfoot, that yielded statistically significant odds ratios in this condition.

Similarly, in the closed eyes condition, compared to the control group, the HAM/TSP group was 6.25 times more likely to have a medium pressure value of 60% on the forefoot and 4.76 times more likely to have a maximum pressure value of 65% on the forefoot. Finally, the HAM/TSP group was 9 times less likely to have a medium pressure value of 40% on the hindfoot and 4.34 times less likely to have a maximum pressure value of 40% on the hindfoot. Also, the reported percentages were the highest that yielded statistically significant odds ratios in closed eyes condition.

## Discussion

The results of this work indicate that HTLV-1 infection caused a displacement of the center of pressure of the body weight towards. To avoid falling, the pressure on the forefoot was increased. The body weight load balance of HAM/TSP patients was remarkably displaced from the hindfoot to the forefoot, while HTLV-1 patients showed a relevant balance displacement to the forefoot but normal pressure loads on the hindfoot. The pressure values of HTLV-1 patients were intermediate between the values measured in controls and HAM/TSP patients, suggesting a gradual worsening of these HTLV-1 infection-related complications towards the more severe phenotype observed in HAM/TSP patients.

Zunt et al. [28] used an objective evaluation of spasticity to quantify the muscle tone in the lower limbs of Peruvian HTLV-1 seropositive women with no history of spasticity. The authors observed subclinical signs of an increased muscle tone in a large fraction of HTLV-1 patients and suggested that HTLV-1 could affect the central nervous system in early stages of the infection. Dias et al. [29] used a modified Ashworth scale to evaluate the muscle tone of HAM/TSP patients and the Tinetti Performance—Oriented Mobility Assessment scale to evaluate their balance. These authors also found a slightly increased muscle tone in three muscle groups and a considerable fraction of patients at risk of falling. Cunha et al. and Macêdo et al. [25, 30] evaluated Brazilian populations in areas of high HTLV-1 infection prevalence and investigated the correlation between postural changes and the patterns of body weight distribution on the foot in patients infected by HTLV-1 [25, 30]. The main postural changes observed were the displacement of head and body positions towards the front, hip extension, and reduction in the ankle angle. Cunha et al. also verified that the shift in the body weight balance towards the forefoot occurred bilaterally, and the authors concluded that the postural changes were responsible for these changes in the body weight distribution [30]. In the present study, changes in the body weight balance were found in both groups of HTLV-1 seropositive patients. HTLV-1 patients showed high pressure loads at the hallux and first metatarsus, whereas HAM/TSP patients had high pressure loads in these areas and also on the remaining toes. This finding may indicate a gradual transition from the initial stages of HTLV-1 infection, without overt neurological symptoms, to later stages when the disease worsens and neural disturbances emerge, eventually leading to HAM/TSP. The same rationale can explain the results observed in the hindfoot: HAM/TSP patients had an abnormally low body weight distribution in this area, whereas HTLV-1 seropositive patients showed a pressure load pattern similar to the pattern of control individuals. These results support the hypothesis of a progressive shift in the body weight balance: from physiological in healthy controls to mildly altered in HTLV-1 seropositive asymptomatic patients to significantly changed in HAM/TSP patients, as suggested by Zunt et al. [28]. The reports of motor weakness and spasticity caused by damage to the upper motor neurons may account for some of these findings of increased plantar pressure on the forefoot; however, a more detailed investigation of the correlation between spasticity and loss of balance would be necessary.

Tispismana et al. [31] used various clinical scales to evaluate HAM/TSP progression in Peruvian population and found that the HAM/TSP progression was faster in male patients but there was no correlation between disease progression and provirus load. We used Berg balance scale as a functional indicator of the patient’s condition. All HTLV-1 patients had only a low risk of falling (and only minor changes in the body weight load distribution on the foot), whereas half of HAM/TSP patients had a moderate to high risk of falling. This increased risk of falling in HAM/TSP patients could be correlated to the significantly increased body weight load on the forefoot.

These changes may be due to the disease pathophysiology, which causes spasticity, muscular weakness, abnormal joint mobility, proprioception impairment, and muscle shortening [12, 29]. The spasticity observed in HAM/TSP patients is caused by inflammatory damages to the spinal cord, leading to the impairment or loss of the inhibitory mechanisms regulating motor neurons activity [32]. This is a phenomenon commonly associated with muscular weakness, changes in motor behavior, hypereflexia, abnormal posture, Babinski reflex, and impaired gait [6–10,12,13,33]. The postural adjustments in patients with HTLV-1 may be a compensatory mechanism to avoid loss of balance. In this study, we observed that HAM/TSP aggravated the abnormal patterns of body weight load distribution on the foot. Disturbances in proprioception are responsible for important neurological changes, leading to alterations in the position of body and limbs and in the alignment of the center of mass with the base of support [19]. Altered proprioception is frequent in patients infected with HTLV-1 and suffering from HAM/TSP, and it could be one of the factors responsible for the postural changes observed in these patients [16,34,35]. In our study, we observed that the skin tactile sensitivity of different regions on the plantar surface of the foot was altered in both groups of HTLV-1 patients. Diminished tactile sensitivity in specific foot areas could lead the patients to adjust accordingly the pressure loads applied to these areas to maintain the static postural balance. However, impaired proprioceptive sensitivity in the study participants may be in part responsible for the alteration in the body weight distribution that in turn caused the shift in the position of the center of mass leading to posture and balance alterations.

Arnaut et al. used virtual reality to evaluate the balance, pain, and quality of life of patients with HAM/TSP [36]. The authors observed that these individuals had low scores in Berg balance scale and extremely impaired posture and balance, and that treatment did not ameliorate these conditions.

Many of the HTLV-1 patients who develop HAM/TSP need assistance to perform their daily activities (e.g., by using crutches or a wheelchair) because of their physical disability, risk of falling, and reduced quality of life and working ability [25,33,35]. The changes in the body weight distribution observed in this study could be a good predictive parameter identify the patients infected with HTLV-1 who are prone to develop HAM/TSP before the onset of the disease symptoms, thus allowing patients and physicians to take timely preventive measures.

## Conclusions

Changes in plantar pressure caused by modifications in the body weight distribution on the foot were found in both groups of HTLV-1 patients. HTLV-1 asymptomatic patients and HAM/TSP patients had higher pressure loads on the forefoot compared to control individuals. HAM/TSP patients also showed lower pressure loads on the hindfoot. These findings suggest that patients with HTLV-1 may represent a transitional step in the progression from healthy individuals to patients with HAM/TSP, and that baropodometry can be a functional indicator of the neurological status of HTLV-1 infected patients.

## Supporting Information

S1 Database Database of plantar pressures from foot regions in closed and open eye conditions. For each foot region, we showed the database from control, HTLV-1 and HAM/TSP groups.
(XLSX)

## References

[1] Uchiyama T, Yodoi J, Sagawa K, Takatsuki K, Uchino H. Adult T-cell leukemia: clinical and hematologic features of 16 cases. Blood. 1977;50: 481–492. 301762

[2] Kaplan JE, Osame M, Kubota H, Igata A, Nishitani H, Maeda Y, et al The risk of development of HTLV-I-associated myelopathy/tropical spastic paraparesis among persons infected with HTLV-I. J Acquir Immune Defic Syndr. 1990;3: 1096–1101. 2213510

[3] Orland JR, Engstrom J, Fridey J, Sacher RA, Smith JW, Nass C, et al Prevalence and clinical features of HTLV neurologic disease in the HTLV Outcomes Study. Neurology. 2003;61: 1588–1594. 14663047 10.1212/01.wnl.0000096011.92542.da

[4] van Sickels NJ, McLellan SLF. Human T-Lymphotropic Virus Types I and II infection In: Magill AJ, Ryan ET, Hill D, Solomon T, editors. Hunter's tropical medicine and emerging infectious diseases. 9th ed Philadelphia: Saunders; 2013 pp. 375.

[5] Osame M, Usuku K, Izumo S, Ijichi N, Amitani H, Igata A, et al HTLV-I associated myelopathy, a new clinical entity. Lancet. 1986;3: 1031–1032. 10.1016/s0140-6736(86)91298-5 2871307

[6] Vernant JC, Maurs L, Gessain A, Barin F, Gout O, Delaporte JM, et al Endemic tropical spastic paraparesis associated with human T-lymphotropic virus type I: a clinical and seroepidemiological study of 25 cases. Ann Neurol. 1987;21: 123–130. 3030190 10.1002/ana.410210204

[7] Bhigjee AI, Kelbe C, Haribhai HC, Windsor IM, Hoffmann MH, Modi G, et al Myelopathy associated with human T cell lymphotropic virus type I (HTLV-I) in natal, South Africa. A clinical and investigative study in 24 patients. Brain. 1990;113: 1307–1320. 2245298 10.1093/brain/113.5.1307

[8] Araujo AQ, Alfonso CR, Schor D, Leite AC, Andrada-Serpa MJ. Clinical and demographic features of HTLV-1 associated myelopathy/tropical spastic paraparesis (HAM/TSP) in Rio de Janeiro, Brazil. Acta Neurol Scand. 1993;88: 59–62. 8372632 10.1111/j.1600-0404.1993.tb04188.x

[9] Gotuzzo E, Cabrera J, Deza L, Verdonck K, Vandamme AM, Cairampoma R, et al Clinical characteristics of patients in Peru with human T cell lymphotropic virus type 1-associated tropical spastic paraparesis. Clin Infect Dis. 2004;39: 939–944. 15472843 10.1086/423957

[10] Lannes P, Neves MAO, Machado DCD, Miana LC, Silva JG, Bastos VHV. Paraparesia espástica tropical—Mielopatia associada ao vírus HTLV-1: possíveis estratégias cinesioterapêuticas para a melhora dos padrões de marcha em portadores sintomáticos. Revista Neurociências. 2006;14: 153–160.

[11] Unsong O, Jacobson S. Treatment of HTLV-I-Associated Myelopathy / Tropical Spastic Paraparesis: towards rational targeted therapy. Neurol Clin. 2008;26: 781–797. 10.1016/j.ncl.2008.03.008 18657726 PMC2610848

[12] Yamano Y and Sato T. Clinical pathophysiology of human T-lymphotropic virus-type 1-associated myelopathy/tropical spastic paraparesis. Front Microbiol. 2012;3: 389 10.3389/fmicb.2012.00389 23162542 PMC3494083

[13] Satou Y, Matsuoka M. Virological and immunological mechanisms in the pathogenesis of human T-cell leukemia virus type 1. Rev Med Virol. 2013;23: 269–280. 10.1002/rmv.1745 23606621

[14] Verdonck K, González E, Van Dooren S, Vandamme AM, Vanham G, Gotuzzo E, et al Human T-lymphotropic virus 1: recent knowledge about an ancient infection. Lancet Infect Dis. 2007;7: 266–281. 17376384 10.1016/S1473-3099(07)70081-6

[15] Carneiro-Proietti AB, Ribas JG, Catalan-Soares BC, Martins ML, Brito-Melo GE, Martins-Filho OA, et al Infection and disease caused by the human T cell lymphotropic viruses type I and II in Brazil. Rev Soc Bras Med Trop. 2002;35: 499–508. 12621671 10.1590/s0037-86822002000500013

[16] Ribas JG, Melo GC. Human T-cell lymphotropic virus type 1(HTLV-1)-associated myelopathy. Rev Soc Bras Med Trop; 2002;35: 377–384. 12170334 10.1590/s0037-86822002000400015

[17] Santos FLN, Lima FWM. Epidemiologia, fisiopatogenia e diagnóstico laboratorial da infecção pelo HTLV-I. J Bras Patol Med Lab. 2005;41: 105–116.

[18] Tinetti ME, Speechley M, Ginter SF. Risk factors for falls among elderly persons living in the community. N Engl J Med. 1988;319: 1701–1707. 3205267 10.1056/NEJM198812293192604

[19] Shumway-Cook A, Horak FB. Assessing the influence of sensory interaction of balance. Suggestion from the field. Phys Ther. 1986;66: 1548–1550. 3763708 10.1093/ptj/66.10.1548

[20] Kitaoka HB, Lundemberg A, Luo ZP, An KN. Kinematics of the normal arch of the foot and ankle under physiologic loading. Foot Ankle Int. 1995;16(8):492–9. 8520662 10.1177/107110079501600806

[21] Donaghue VM, Veves A. Foot pressure measurement. Orthop Phys Ther Clin N Am. 1997; 6: 1509–16.

[22] Vianna DL, Greve JMD. Relação entre a mobilidade do tornozelo e pé e a magnitude da força vertical de reação do solo. Rev. bras. fisioter., 2006; 10 (3): 339–345.

[23] Monteiro, VA. Ergonomia, design e conforto no calçado feminino. Rio de Janeiro, PUCRJ, 1999. Dissertação (mestrado). Pontifícia universidade católica do Rio de Janeiro—Departamento de artes, Rio de Janeiro, 1999.

[24] Rosário JLP. A review of the utilization of baropodometry in postural assessment. Journal of Bodywork & Movement Therapies, 2014; 18: 215–219. 24725789 10.1016/j.jbmt.2013.05.016

[25] Macêdo MC, Baptista AF, Castro-Filho BG, Duarte EF, Patrício N, Kruschewsky RA, et al Postural profile of individuals with HAM/TSP. Braz J Med Hum Health. 2013;2: 99–110.

[26] WHO. Report of the scientific group on HTLV-1 and associated diseases, Kangoshima, Japan. Virus disease Human T-lymphotropic virus type 1, HTLV-1. Wkly Epidem Rec. 1989: 382–383.

[27] Ayres M, Ayres M Jr, Ayres DM, Santos AAS. BioEstat3.0. Aplicações estatísticas nas áreas das ciências bio-médicas. 3rd ed Belém: Sociedade Civil Mamirauá / MCT-CNPq / Conservation International; 2003.

[28] Zunt JR, Alarcón JOV, Montano S, Longstreth WT, Price R, Holmes KK. Quantitative assessment of subclinical spasticity in human T-cell lymphotropic virus type I infection. Neurology. 1999; 53 (2): 386–390. 10430431 10.1212/wnl.53.2.386 PMC2678023

[29] Dias GAS, Yoshikawa GT, Koyama RVL, Fujihara S, Martins LCS, Medeiros R, Quaresma JAS, Fuzii HT. Neurological manifestations in individuals with HTLV-1-associated myelopathy/tropical spastic paraparesis in the Amazon. Spinal Cord. 2015; 1–4. 26169165 10.1038/sc.2015.112

[30] Cunha EFD, Patrício NA, Macedo MC, Sena C, Kruschewsky R, Castro Filho BG, et al Postural profile of patientes with HAM/TSP: computerized and baropodometric assessment. Braz J Med Hum Health. 2013;1: 19–33.

[31] Tipismana M, Verdonck K, González E, López G, Clark D, Gotuzzo E. Subacute progression of HTLV-1 associated myelopathy/tropical spastic paraparesis in Peru: a report of 20 cases. Infectious diseases of the nervous system: pathogenesis and worldwide impact. Paris, France. 2008; 10–13 September.

[32] Osame M. Pathological mechanism of human T-cell lymphotropic virus type 1-associated myelopathy (HAM/TSP). J Neuro Virol. 2002; 8: 350–364. 10.1080/13550280260422668 12402162

[33] Franzoi AC, Araújo AQ. Disability and determinants of gait performance in tropical spastic paraparesis/HTLV-I associated myelopathy (HAM/TSP). Spinal Cord. 2007;45: 64–68. 16568145 10.1038/sj.sc.3101919

[34] Souza A, Tanajura D, Toledo-Cornell C, Santos S, de Carvalho EM. Immunopathogenesis and neurological manifestations associated to HTLV-1 infection. Rev Soc Brasil Med Trop. 2012;45: 545–552. 10.1590/s0037-86822012000500002 23152334

[35] Reiss DB, Freitas GS, Bastos RHC, de Souza MA, Horiguchi CLF, Martins ML, et al Neurological outcomes analysis of HTLV-1 seropositive patients of the Interdisciplinary Research HTLV Group (GIPH) cohort, Brazil. Retrovirology. 2014;11(Suppl 1): P51.

[36] Arnaut VACO, Macêdo MC, Pinto EB, Baptista AF, Castro-Filho BG, Sá KN. Virtual reality therapy in the treatment of HAM/TSP individuals. Physical Therapy & Research. 2014; 4: 99–106.

